# Feed, Microbiota, and Gut Immunity: Using the Zebrafish Model to Understand Fish Health

**DOI:** 10.3389/fimmu.2020.00114

**Published:** 2020-02-05

**Authors:** Adrià López Nadal, Wakako Ikeda-Ohtsubo, Detmer Sipkema, David Peggs, Charles McGurk, Maria Forlenza, Geert F. Wiegertjes, Sylvia Brugman

**Affiliations:** ^1^Cell Biology and Immunology Group, Wageningen University and Research, Wageningen, Netherlands; ^2^Aquaculture and Fisheries Group, Wageningen University and Research, Wageningen, Netherlands; ^3^Laboratory of Animal Products Chemistry, Graduate School of Agricultural Science, Tohoku University, Sendai, Japan; ^4^Microbiology, Wageningen University and Research, Wageningen, Netherlands; ^5^Skretting Aquaculture Research Centre, Stavanger, Norway

**Keywords:** zebrafish, immunity, prebiotics, probiotics, microbiota, intestine, gut

## Abstract

Aquafeed companies aim to provide solutions to the various challenges related to nutrition and health in aquaculture. Solutions to promote feed efficiency and growth, as well as improving the fish health or protect the fish gut from inflammation may include dietary additives such as prebiotics and probiotics. The general assumption is that feed additives can alter the fish microbiota which, in turn, interacts with the host immune system. However, the exact mechanisms by which feed influences host-microbe-immune interactions in fish still remain largely unexplored. Zebrafish rapidly have become a well-recognized animal model to study host-microbe-immune interactions because of the diverse set of research tools available for these small cyprinids. Genome editing technologies can create specific gene-deficient zebrafish that may contribute to our understanding of immune functions. Zebrafish larvae are optically transparent, which allows for *in vivo* imaging of specific (immune) cell populations in whole transgenic organisms. Germ-free individuals can be reared to study host-microbe interactions. Altogether, these unique zebrafish features may help shed light on the mechanisms by which feed influences host-microbe-immune interactions and ultimately fish health. In this review, we first describe the anatomy and function of the zebrafish gut: the main surface where feed influences host-microbe-immune interactions. Then, we further describe what is currently known about the molecular pathways that underlie this interaction in the zebrafish gut. Finally, we summarize and critically review most of the recent research on prebiotics and probiotics in relation to alterations of zebrafish microbiota and immune responses. We discuss the advantages and disadvantages of the zebrafish as an animal model for other fish species to study feed effects on host-microbe-immune interactions.

## Zebrafish as a Model for Immunity

In late 1960s, the Hungarian molecular biologist George Streisinger obtained zebrafish (*Danio rerio*) to investigate molecular mechanisms applying forward genetics in a vertebrate model [reviewed in (1)]. Initially, researchers used zebrafish to study developmental biology followed by the employment of zebrafish in numerous other fields. Among these, zebrafish stood-out as a model to study immunity due to the high presence (~70%) of human orthologous genes in the zebrafish genome (2) and its intrinsic characteristics. Zebrafish are small (<5 cm), highly prolific (200–300 new progeny per week) and fast growing compared to mice. Zebrafish develop *ex-utero* which, combined with the embryos' transparency, enables investigation of ontogeny *in vivo* from an early time point in development [reviewed in (3)]. Moreover, the use of transgenic fish facilitates *in vivo* visualization of specific immune cell populations such as neutrophils (4) based on expression of the neutrophil-associated enzyme myeloperoxidase (5) using fluorescent microscopy. In addition, their well-annotated genome eased the generation of mutant zebrafish lines, some of which contributed to elucidate immune gene functions [reviewed in (3)]. In the last decade, genome editing techniques based on Zinc finger nuclease [reviwed in (6)], TALENs (7) and the highly successful CRISPR-Cas technique (8, 9) changed the speed at which single gene functions can be addressed in this model organism. Currently gene insertion still appears more challenging than gene knock-out, something that will undoubtedly change in the near future (10). Zebrafish characteristics combined with these unique research tools established these small cyprinids as an important animal model to study immune processes and underlying molecular mechanisms.

## Zebrafish Intestine: Structure, Function, and Microbiota

Zebrafish do not have a stomach and their digestive tract is anatomically divided into separate sections: the mouth, the esophagus, three gut segments (anterior, middle, and posterior) and the anus. The zebrafish esophagus is connected with the anterior gut segment, where the nutrient absorption predominantly occurs due to a high presence of digestive enzymes. Nutrient uptake gradually diminishes from the anterior to the posterior gut segments. Ion transport, water reabsorption, fermentation processes as well as certain immune functions occur in the middle and posterior gut segment (11, 12). Wang et al. investigated the gene expression of the adult zebrafish gut and compared it to the gut of mice which is anatomically divided into: mouth, esophagus, stomach, three small intestine sections [duodenum, jejunum, and ileum), cecum, large intestine, rectum and anus (13)]. In this study the zebrafish gut was divided into equal-length segments (called S1–S7, from anterior to posterior) and, based on subsequent transcriptomic analysis, regrouped into three main segments: S1–S5, S6, and S7 corresponding to small and large murine gut (14). Subsequently, Lickwar et al. performed transcriptomics on adult intestinal epithelial cells (IECs) from zebrafish, stickleback, mouse and human (15). They specified that the segments S1-S4 of the zebrafish gut presented 493 highly expressed genes from which 70 were also upregulated in the mouse anterior gut (duodenum and ileum-like segments). Next to this, the authors found a core set of genes present in all vertebrate IECs as well as conservation in transcriptional start sites and regulatory regions, independent of sequence similarity (15).

Besides all the similarities described above, there are clear anatomical differences between zebrafish and the murine digestive tract. Zebrafish do not have a stomach, intestinal crypts, Peyer's patches nor Paneth cells [reviewed in (16)]. In addition, there are dissimilarities in feeding habits, environmental conditions, body sizes and/or specific metabolic requirements. The fact that for instance, lipid metabolism is regulated by similar gut segments between zebrafish and mouse does not imply homology since their metabolism differs greatly: i.e., zebrafish do not have brown fat (13). Still it remains striking that IECs of different species are more similar in gene expression and regulation (regardless of species intestinal anatomy or feeding habits) than different cell types of the same species (15). The evidence that gene expression and regulation of this expression in the gut is so highly conserved between species suggests the potential of zebrafish as a valid model for other fish species such as other cyprinids or salmonids when investigating intestinal function.

It has been shown in mice that colonization of the gut with specific microbes induces immune system function. For example, colonization of germ-free (GF) mice with segmented filamentous bacteria induced activation of CD4+ T cells as well as IgA production (17). Rawls et al. generated a GF zebrafish larval model to study the function of the gut microbiota (18). Using this model they examined the effect of colonization on the host transcriptional response (6 dpf -days post fertilization- larvae) by DNA microarray analysis. Similarly to mice or humans, microbiota-associated gene expressions clustered in several canonical pathways mainly related to four physiological functions: epithelial cell turn-over, nutrient metabolism, xenobiotic metabolism, and innate immune responses (18). In mammals, microbiome colonization may occur during birth (19) or prenatally in the womb (20). In zebrafish, microbiome colonization is thought to occur at hatching although vertical transmission of microbiome components during oviposition has also been suggested (21). Recently, the colonization cycle of microbial species into the gut of zebrafish larvae has been studied in more detail using several generations of GF zebrafish larvae mono-associated with *Aeromonas veronii* (22). The colonization cycle was found to be divided in four steps: (1) immigration of environmental microbes into the fish, (2) gut adaptation of such microbes, (3) microbe emigration from the host to the environment, and (4) environmental adaptation of the microbes. Both environmental and host gut microbial adaptation were assessed by microbial growth rate, abundance and persistence within the gut or the environment. When comparing four evolved isolates (undergone multiple cycles through the host) and the ancestral strain the authors observed that the evolved isolates were more abundantly present in the fish gut, emphasizing the role of immigration and further adaptation of species into the zebrafish gut.

Earlier colonization studies showed that immigration into the host and gut adaptation are found to be time-specific for each microbe: γ-Proteobacteria were highly abundant in environmental samples as well as in the gut of zebrafish larvae while β-Proteobacteria were mostly abundant in environmental samples and in the gut of juvenile zebrafish, indicating a delayed colonization by certain species of β-Proteobacteria after initial exposure (23). Further research may clarify the specific species involved in the colonization process and whether the colonization delay is due to low microbe immigration to or adaptation to the host gut. During colonization, two major microbial shifts in colonization of zebrafish were described: a first shift at 10 dpf from embryo to larvae and a second shift between 35 and 75 dpf, from juvenile to early adult (23). During the first shift at 10 dpf some individuals had high taxa an richness samples (resembling embryos) while others showed low taxa richness and diversity (resembling juveniles). This distribution could be the result of different developing speed among the larvae. Since feeding generally commences at 6 dpf and zebrafish larvae actively hunt for the (live) feed some fish grow and develop faster than others. In support of the zebrafish observations, studies in other fish species also describe an age-dependent decrease in species density and diversity of the gut microbial community from larval to adult stages [reviewed in (24)]. The embryo-to-larva shift could be due to the consumption of exogenous feed (Paramecium) and the juvenile-to-early-adult shift could be due to physiological processes such as sexual maturation (23). Nonetheless, it cannot be excluded that microbiota may adapt and expand due to certain feed components or that the live feed itself brings along microbes and microbial analysis of feed samples could further clarify gut colonization dynamics. Most significantly, so far a putative contribution of a maturing immune system regarding microbiota composition has hardly been addressed in zebrafish.

Larval zebrafish have functional and well-developed organs but their immune system is not completely mature yet. Adaptive immune maturation in zebrafish is an active research topic within the scientific field. In a relatively small study, we showed that T cells control Proteobacteria *(Vibrio)* abundance in the zebrafish gut, providing evidence that like in mice the adaptive immune system plays a role in shaping the microbiota composition (25). T cells are present in the thymus by 4 dpf as shown by using CD4-1:mCherry transgenic zebrafish (26) and CD8a+ antibody staining (27). It was shown that T cells egress from the thymus as early as 10 dpf. This suggests that from that time point onwards systemic adaptive responses could be mounted in the zebrafish. However, more in depth studies on the exact timing (the variability thereof) and functionality of these thymic emigrants are warranted.

After the initial colonization period, important for both host and microbe development, the microbiota is believed to enter a stable state. Comparison of gut microbiota of wild-caught zebrafish and zebrafish raised in two separate laboratory facilities revealed that there is a shared so-called core gut microbiota (23, 28). High quality 16S rRNA gene analysis showed common and abundant bacterial groups represented by 21 operational taxonomic units (OTUs), dominated by members of the Proteobacteria phylum (genera *Aeromonas* and *Shewanella*) followed by Fusobacteria or Firmicutes (class Bacilli), Actinobacteria and Bacteroidetes phyla (28).

In conclusion, all organisms on earth are colonized with bacterial species from their environment. The host and colonizing microbes adapt to ensure fitness of both the host and microbiota. It is important to realize that only performing colonization studies using zebrafish larvae may not represent the complete picture. Especially the maturation of the host immune system can have a profound effects on shaping the intestinal microbiota and, therefore, extrapolation of larval results to juveniles or adults should be carefully examined. Nonetheless, the fact that zebrafish can be reared GF and are still optically transparent at 10 dpf together with the possibility of transgenesis of immune cell populations make zebrafish a very powerful organism to study the timing of microbial colonization and immune system maturation.

## Shaping the Microbiota: Environmental and Host Factors

Microbes can establish symbiotic relationships with their host by, for instance, facilitating nutrient digestion of diets. Host (biotic) and environmental (abiotic) factors play a role in the modulation of the (intestinal) microbiota. For example, zebrafish larvae exposed to naturally found concentrations of antibiotics together with an antinutritional factor (soy saponin) showed an increased neutrophil recruitment in the gut as well as dysbiosis in the overall microbiome composition (29). A meta-analysis of 16S rRNA gene sequence data from 25 individual fish gut communities (30) integrated five already published zebrafish data-sets (28, 31). Microbial intestinal communities from different species clustered together and separately from environmental samples. Within the intestinal microbial cluster different gut bacterial communities exist depending on trophic level (herbivores, carnivores, or omnivores), habitats (saltwater, freshwater, estuarine, or migratory fish), and sampling methods (30). Taking the observations together, the symbiotic process between host and bacteria is highly conserved and partly depends on diet and natural habitat.

So which host mechanisms influence the gut microbiota composition? In order to study to what extend the gut selects the microbial community, GF mice were colonized with gut microbiota of conventionally-raised (CONV) zebrafish and vice-versa, GF zebrafish were colonized with gut microbiota of CONV mice. The mouse microbiota generally contains a higher proportion of Firmicutes and Bacteroides compared to the zebrafish microbiota which is dominated by Proteobacteria. Interestingly, after transfer of the mouse microbiota into GF zebrafish, the relative abundance of the Proteobacteria increased toward a microbiota composition of zebrafish. Vice-versa, when zebrafish microbes (dominated by Proteobacteria) were transferred to mice recipient the Firmicutes from this zebrafish content flourished up to >50% compared to the Firmicutes abundance of 1% in original zebrafish microbiota (31). Therefore, it seems that the host gut environment shapes the microbiota.

The immune system is part of this host gut environment. For example, zebrafish gut macrophages can shape the microbiota via interferon regulatory factor *irf8*. Adult *irf8*-deficient zebrafish displayed a reduced number of macrophages (mpeg1.1 promoter), presented reduced *c1q* genes expression (*c1qa, c1qb, c1qc*, and *c1ql*) and severe dysbiosis (Fusobacteria, α- and γ-Proteobacteria diminished in favor of δ-Proteobacteria) compared to controls. Downregulation of *c1q* genes may imply an ineffective complement system which could contribute to the observed dysregulation of commensal microbiota. Restauration of *irf8* expression reversed *c1q* genes expression and the levels of commensal microbes (32). However, a recent study showed that the mpeg1.1 promoter is not only marking macrophages but also phagocytic B lymphocytes in adult zebrafish (33). This might indicate that B cells might also play a role in shaping the microbiota.

In addition to the influence of the fish innate immune system on shaping the microbial communities, there is evidence that the adaptive immune system also plays a role in this process. Adult wild-type zebrafish displayed a decreased abundance of Proteobacteria (*Vibrio)* compared to zebrafish lacking adaptive immunity (rag1-/-), indicating that the innate immune system alone cannot fully regulate all members of the microbiota in the gut. Also, adoptive transfer of T and non-T cells (B and NK-like cells) from wild-types to rag1-/- fish showed that transfer of T cells, but not B/NK-like cells, in the rag1-/- fish diminished *Vibrio* spp. outgrowth 1 week after transfer, suggesting that T cells could regulate the abundance of certain intestinal microbial species. Furthermore, the lack of adaptive immune response together with altered microbiota induced an inflamed state in the gut of aged zebrafish (14 weeks post feralization): *il-1*β and *cxcl2-l2* were upregulated and *il10, ifn*γ, and *il17f2* downregulated compared to controls. These aged rag1-/- zebrafish developed dropsy (edema caused by bacterial infection) or became anorexic, confirming the physiological effects of an absence of adaptive immunity and possibly a dysregulated microbiota (25). Others also tested the contribution of the adaptive immune system to gut microbiota in adult zebrafish. In this study, rag1-/- or wild-type zebrafish were either housed separately or were co-housed. In segregated genotypes, rag1-/- microbial communities differed from that of wild-types, suggesting a selective pressure of the adaptive immune system. However, such effect was lost when rag1-/- and wild-type zebrafish were housed together (34). This study suggested that housing could have more influence on microbial diversity than (the absence of the) adaptive immunity. The observation seems to contradict an earlier meta-analysis where different rearing conditions did not result in phylogenetically divergent gut microbiota although cohousing of distinct genotypes was not included in their study (30). Even though the exact extent to which the host immune system affects the microbiota is not completely elucidated, the aforementioned studies (25, 31, 32, 34) suggest selective pressures of the innate and adaptive immune system on the composition of the host gut microbiota.

Contrary to the putative selective pressure of the gut immunity on the microbiota, chance and random distribution (neutral model) was also investigated as explanation for the initial/early assembly of the zebrafish gut microbial community (35). Non-neutral processes, such as immune system or feed could become more important for microbial modulation at older stages. Gut bacterial communities in zebrafish could be modulated mostly by ecological dynamics outside of the host, on a broader scale (35, 36). Although microbial ecology processes outside the host certainly play a role in the assembly of the host-gut microbiota, it seems unlikely that chance and random microbial dispersion could vastly explain the similarities of gut microbial compositions across species (30). The fact that gut microbial communities of mammals and fish cluster together suggests that specific pressures to the intestinal environment shape the intestinal microbiota. The earlier mentioned colonization cycle proposed by Robinson et al. (22) already takes into account a broader perspective of the environmental ecology including extra- and intra-host factors, such as gut adaptation of the microbes, but only non-fed larvae were analyzed. Taken together these observations, it is highly probable that the intestinal microbiota is, at least partly, modulated by the innate and adaptive host-immune system.

## Microbe-Host Interaction in Zebrafish Intestine: Molecular Immune Mechanisms

**Figure 1:**
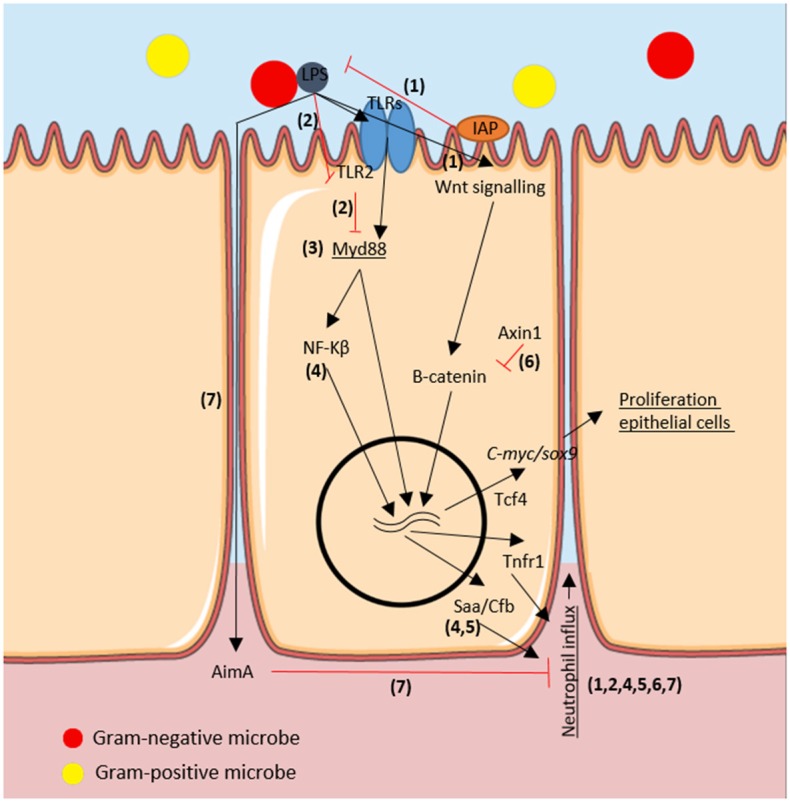
Immuno-modulatory molecular pathways regarding the microbe-host interaction in the epithelium of the zebrafish intestine. We depicted the molecules involved in the proliferation of epithelial cells and in the neutrophil influx as a host-responses to microbiota in the zebrafish gut. In black arrows activation processes, in red inhibition processes. Genes are in italics and host-associated responses are underlined. Numbers correspond to articles proving such molecular interactions: 1: Bates et al. (37); 2: Koch et al. (38); 3: Troll et al. (39) 4: Kanther et al. (40), 5: Murdoch et al. (41), 6: Cheesman et al. (42), and 7: Rolig et al. (43).

The host gut exerts selective pressure on the microbiota (reviewed in the section above), which in turn influences host immune responses. In Figure 1, we summarized the host-microbe molecular pathways in the zebrafish gut cells. Commensal gram-negative microbes produce low quantities of lipopolysaccharide (LPS) which activate intestinal alkaline phosphatase (Iap) (44). Iap is an endogenous protein located in the apical intestinal epithelium and secretes surfactant-like particles to the intestinal lumen (45). Activated Iap counteracts LPS-associated intestinal inflammation, as quantified by neutrophil infiltration in the gut of zebrafish larvae (37). In mammals, after Toll like receptor (TLR)-microbial recognition and Myd88 adaptor protein activation, a downstream signaling cascade follows, including nuclear factor κ-light-chain-enhancer of activated B cells (NF-κB) signal transduction to the nucleus [reviewed in (46); and in (47)].

Recently, a TLR2-Myd88-dependent transcriptional feedback mechanism was described upon microbial colonization by using *myd88* deficient zebrafish larvae (38). The proposed mechanism involves microbial stimuli being recognized by TLR2 and partly suppress *myd88* but enabling enough *myd88* transcriptional activity to possibly induce protective mucin secretion in the apical intestinal epithelium. However, downstream TLR-*myd88* induction of mucin has only been demonstrated in *ex-vivo* mice experiments (48) and not yet in zebrafish. In GF zebrafish, TLR2 cannot suppress *myd88* expression and its elevated levels leads to stimulation of activator protein 1 (AP-1) transcription factors, which resulted in an overall increase in leukocytes (macrophages) in the gut (38). Nonetheless, GF zebrafish did not show enhanced inflammation as could be expected from AP-1 over-expression. Thus, other mechanisms perhaps absent in larval stages–i.e., adaptive immunity- must be involved in myd88 regulation. Knock-out myd88-/- juveniles or adult zebrafish could be used to further investigate the role of adaptive immunity in regulating microbe-host interaction.

In line with the observation that Myd88 is a key regulator of host-microbe interaction in the gut of larval zebrafish, microbiota determined secretory or absorptive differentiation of IECs via inhibiting Myd88-Notch signaling (39). Notch signaling is a crucial mechanism for intestinal stem cell differentiation into secretory intestinal cells in zebrafish (49). The study focused more on the downstream Myd88 signaling rather than on the recognition of the microbes via TLRs. TLRs have been thoroughly studied in zebrafish [reviewed in (50)] yet to our knowledge there are no studies showing a direct link of feed components to subsequent TLR-myd88-Notch signaling and increased secretory fate of IECs (Goblet cell differentiation) via changes in the microbiota. In the future, several TLR knock-out zebrafish could be engineered to understand how specific feed components and/or the microbiota trigger relevant molecular pathways.

Single microbial species can also influence the zebrafish larval immune system. Gram-negative *Pseudomonas aeruginosa* stimulated NF-κB-dependent expression of innate immune genes such as complement factor b (*cfb)* and serum amyloid a (*saa)* which enhanced neutrophil influx (40). In a recent article, *saa*-deficient zebrafish displayed aberrant neutrophil responses to wounding but increased clearance of pathogenic bacteria. Interestingly, *saa* function depended on microbial colonization of GF individuals. To prove that *saa* produced in the gut can systemically affect neutrophil recruitment, they created a transgenic zebrafish expressing *saa* specifically in IECs by using the *cldn15la* promoter fragment to drive mCherry fluorescence, located in the IECs. *Saa* produced in the gut in response to microbiota systemically prevented excessive inflammation (tested by tail amputations) as well as reduced bactericidal potential and neutrophil activation (41). Thus, besides the aforementioned functions (38, 39), Myd88 activation after TLR-microbial recognition orchestrates neutrophil migration to inflamed tissues as previously shown by Kanther et al. (40) and also pathogenic bacterial clearance in a *saa*-dependent manner (41) in zebrafish larvae in response to microbiota.

Further molecular pathways have been studied by generating specific gene mutations in zebrafish, such as a*xin1. Axin1* mutant zebrafish showed upregulated Wnt signaling and β-catenin protein levels (42). It was previously shown in mice that β-catenin accumulates in the cytoplasm and, at a threshold concentration, translocates to the nucleus where (with cofactors such as intestine-specific transcription factor Tcf4) it switches on expression of pro-proliferative genes like *c-myc* or *sox9* (51, 52). Induction of *c-myc* and *sox9* in turn increases IEC proliferation. Similarly, *axin1* mutant zebrafish showed increased cell proliferation in the intestine but not when *axin1* mutant zebrafish were reared GF, indicating that the microbiota triggers this increased cell proliferation, confirming earlier results showing increased epithelial turn-over upon microbial colonization (18). Interestingly, mono-association of resident bacteria *Aeromonas veronii* was enough to increase intestinal cell proliferation in *axin1* mutant zebrafish by the same mechanisms: upregulating Wnt signaling and β-catenin protein expression. It can be concluded that the microbiota plays a role in the proliferation of epithelial cells in the zebrafish gut during microbial colonization via two mechanisms: TLR recognition with Myd88 downstream signaling and Wnt signaling with β-catenin protein accumulation and pro-proliferative gene activation (42). Increased intestinal cell turnover in the developing zebrafish larvae may be beneficial for the host to renew damaged epithelial cells and to shed potentially pathogenic bacteria attached to the epithelium.

To quantify host immune responses to multi-species rather than mono-association, a species quantitative model was created. Two variables were assessed in the zebrafish larvae model: the neutrophil response to individual strains and the absolute abundances of community members. Specific microbes, regardless of their relative abundances, played a major role in the neutrophil influx. GF zebrafish were colonized with different species (*Aeromonas, Vibrio*, and *Shewanella*) and neutrophil influx into the gut was investigated. *Shewanella* partly inhibited the *Vibrio* induction of neutrophil influx in the gut via cell-free supernatant (CFS). However, *Shewanella* CFS did not alter neutrophil influx in combination with *Aeromonas* mono-association (53). This study stresses the fact that mono-association experiments may be important to understand molecular mechanisms, however they may not reflect the *in vivo* situation where microbial species affect each other. Here, the authors used zebrafish larvae and neutrophil influx as the immune parameter, it would be interesting to see effects on other immune mediators, such as eosinophils which are abundantly present in the zebrafish gut. Although the knowledge of immunomodulatory factors produced by fish gut microbiota is limited, a recent study discovered a unique protein AimA (“Aeromonas immune modulator”) secreted by *Aeromonas veronii*, which benefit both host and microbe. While AimA protects the host by preventing chemically and bacterially-induced intestinal inflammation, it protects *A. veronii* from host immune response and enhances colonization (43). Further studies are needed to understand how specific bacterial species and their associated secreted molecules are involved in overall immune modulation in the zebrafish intestine and systemically. For further reading on the modulation of innate immunity to commensal bacteria, we refer to a recently published review of Murdoch and Rawls (54) and for a more extensive review on hematopoiesis in the developing zebrafish to the review of Musad and coworkers (55).

## Impact of Prebiotics and Probiotics on the Zebrafish Microbiota and Gut Immunity

In their natural environment, adult zebrafish eat zooplankton and insects. Analysis of the zebrafish gut content also revealed the presence of phytoplankton, spores and filamentous algae, among others [reviewed in (56)]. There is not a standard diet for zebrafish in captivity and feeding practices include feeding a mixture of live feeds such as rotifers, ciliates, *Artemia nauplii* and formulated dry feeds (57). Supplementary ingredients have been investigated in several commercially relevant fish species in order to increase growth and control aquaculture related diseases (58). More specifically, fish microbial communities may influence the immune system and decrease aquaculture-related diseases [reviewed in (24)]. An overall summary of key operational taxonomic units (OTUs) in various tissues (skin, gut, gills, and digesta) have been associated with fish diseases and infections compared to the wild-type individuals [reviewed in (59)]. The use of zebrafish as experimental model to develop novel feeds for farmed fish has gained interest, especially for the development of prebiotics and probiotics as immune and microbiome modulators [reviewed in (60)]. Although most of the prebiotics and probiotics assure benefits for the host, a careful assessment of their effects remains important, as shown for effects of human probiotics uncovering problematic research design, incomplete reporting, lack of transparency or under-reported safety were described [reviewed in (61)]. In the next section, we review the current literature on the effects of prebiotics and probiotics on the immune system and microbiota of zebrafish.

## Prebiotics

Prebiotics can be defined as non-digestible feed ingredients that have a beneficial effect toward the host by selectively stimulating the growth or the activity of commensal gut bacteria and thus improving host health [reviewed in (62)]. Prebiotics most often consist of small carbohydrate chains that are commercially available as oligosaccharides of glucose (like β-glucans), galactose, fructose, or mannose. The use of prebiotics as immuno-stimulants in farmed fish feed has been reviewed elsewhere (63), however the effect of prebiotics on zebrafish (gut) health and on microbiota composition needs further examination. We summarized such studies in Table 1. Most of the studies have been performed in larval zebrafish and only very few studies have been performed in adults. The most employed prebiotics in zebrafish research were fucoidans (sulphated polysaccharides mainly present in brown algae and brown seaweed), β-glucans (β-D-glucose polysaccharides extracted from cell walls of bacteria and fungi) and sometimes others, such as galactooligosaccharides. It is of note that not much is known about the modulation of the microbiota by prebiotics since most of the reviewed studies only investigated their immune stimulatory effects.

**Table 1:** Summary of prebiotics, probiotics, and synbiont studies performed in zebrafish regarding immunity and microbiota.

	**Specie(s)/strain(s)**	**Zebrafish age**	**Microbiota composition**	**Immune-modulatory effects**	**Other relevant parameters**	**References**
Prebiotic	Fucoidan from *Eklonia cava*	Embryos (not specified)	–	– Reduced the levels of ROS and NO after challenge with LPS and tail cutting	–	(64)
Prebiotic	Fucoidan from *Turbinaria ornata*	3 dpf	–	– Reduced LPS-induced levels of *COX2, iNOS*, and ROS.	−Improved cell viability	(65)
Prebiotic	Fucoidan from *Chnoospora minima*	3 dpf	–	– Reduced LPS-induced levels of *COX2, iNOS*, and ROS.	−Improved cell viability	(66)
Prebiotic	β-glucan from oats	5 dpf	–	– Upregulation of *tnfa, il-1β, il10, il12, defb1, lyz, c-rel*.	−Increased survival after *E. tarda* challenge.	(67)
Prebiotic	β-glucan	4 hpf−6dpf	–	– Upregulation of *tnfa, mpo, trf, lyz*	−Increased survival after *Vibrio anguillarum* challenge	(68)
Prebiotic	Fucoidan from *Cladosiphon okamuranus*	6–9 dpf and adult zebrafish	–Decreased *E coli* and favored Rhizobiaceae and Burkholderiaceae in adults gut but not overall larvae.	– Reduction of *il-1β* but not *cxcl8, il10* nor *tnfb* in the zebrafish adult gut −Increase of *il-1β, il10, tnfb* and *mmp9* in overall larvae.	–	Ikeda-Ohtsubo et al. (in this issue)
Prebiotic	Galactooligosaccharide supplemented in diet (0.5, 1, and 2%)	Adult zebrafish (8 weeks feeding)	–	–Upregulation of *tnfa* and *lyz* −Increase in total immunoglobulin concentration.	–	(69)
Probiotic	2 yeast species: *Debaryomyces* (Db) and *Pseudozyma* (Ps)	2–3 dpf yeast exposure, gut sampling at 14 dpf	−Core microbiota differed from controls. –Reduced Bacteroidetes abundance. –Db increased species richness. –Db increased abundance of *Pediococcus* and *Lactococcus*.	–	–	(70)
Probiotic	*Lactobacillus casei BL23*	From 3 to 25 dpf	–	–Upregulated expression of *il-1β, C3a* and *il-10* after 8 or 24 h post-challenge with *A. hydrophila*.	−Increased survival after *A. hydrophila* challenge	(71)
Probiotic	Yeasts*: Yarrowia lipolytica 242* (Yl242) and *Debaryomyces hansenii 97* (Dh97)	At 4 dpf, 2 h exposure	–Germ-free (GF) larvae and conventionally raised (CONV) larvae.	–Upregulation of *il-1β, c3, tnfa, mpx*, and *il10* in CONV larvae after *V*. *anguillarum* challenge −Pre-treatment with Dh97 and Yl242 prevented gene upregulation in CONV and GF larvae.	–Increased survival of CONV and GF larvae due to yeast after challenge with *V. anguillarum* (GF higher mortality than CONV).	(72)
Probiotic	*Lactobacillus plantarum ST-III* (LAB) and bile salt hydrolase (BSH). Exposure to Triclosan (TCS) alone or with LAB (TL) or BSH (TB).	From 4 hpf to 90 dpf	−Gut microbiota clustered: LAB > Control > TL and BSH > TB > TCS. –TCS shifted the microbiota and when LAB or BSH co-exposed microbiota resembled more to controls.	–LAB and TL reduced malonaldehyde in the gut. –TCS upregulated *NF-kB* and *il-1β, tnfa* expression. –TCS increased CD4+T cells in the lamina propria. –TCS thinned intestinal mucosa, destructed epithelia and increased goblet cells.	–TCS induced fibrosis, increased lipid droplet, increased triglycerides, and total cholesterol concentrations in the liver compared to controls and LAB/TL treated fish.	(73)
Probiotic	15 yeast strains	At 4 dpf, 2 h exposure	–	–Larvae after *V. anguillarum* displayed more neutrophils outside the caudal hematopoietic tissue	–All yeast except Mv15 and Csp9 increased survival after *V. anguillarum* challenge.	(74)
Probiotic	*L. plantarum* WCFS1 and *NA7* and *L. fermentum ATCC9338, NA4*, and *NA6*.	At 5 dpf, 24 h exposure	GF larvae	–NA4 exposure prior to TNBS challenge lowered levels *trfa* and *il-1β* – *Il-10* expression was higher in larvae exposed to NA4	–	(75)
Probiotic	37 commensal or probiotic Gram-positive and Gram-negative bacteria	6–9 dpf	–	–	–Increased survival by *V. parahaemolyticus, E. coli ED1a-sm* and *E. coli MG1655* F' upon *E. ictaluri* infection.	(76)
Probiotic	*Lactobacillus rhamnosus*	96 hfp, 6 and 8 dpf	–Increased the rel. abundance of Firmicutes	–Enlarged enterocytes and microvilli on the apical surface of the epithelium.	–Increased total length and wet weight at 8 dpf.	(77)
Probiotic	B*. coagulans, L. plantarum, L. rhamnosus, Streptococcus thermophilus, Bifidobacterium infantis*.	Adult zebrafish (28 days feeding)	–	–*B. coagulans* and *L. plantarum* reduced the number of Masts cells in the gut after *A. hydrophila* challenge. –*B. coagulans* and *L. plantarum* reduced expression of *tnfa* and *il10* and increased *il-1β* in the gut.	–*B. coagulans* and *L. plantarum* reduced mortality after *A. hydrophila* challenge.	(78)
Probiotic	*Lactobacillus plantarum*	Adult zebrafish (30 days feeing)	–*L. plantarum* clustered gut microbiota independently –Reduced rel. abundance of Vibrionaceae, Pseudoalteromonadaceae, and Leuconostrocaceae and increased Lactobacillaceae, Stenotrophomonas, and Catenibacterium.	–Not clear effect of *L. plantarum*	–Upregulated canonical pathways related with energy metabolism and vitamin biosynthesis.	(79)
Probiotic	*Lactobacillus rhamnosus*	Adult fish (10 days feeding)	–	–Upregulated expression of *il1b, tnfa*, and *becn1* in the gut.	–	(80)
Probiotic	8 probiotic strains were lyophilized and mixed with a commercial diet	Adult fish (30 days feeding)	–	–Downregulated *casp4* and *baxa* and upregulated *bcl2a* in the gut. – Upregulated *il-1β, tnfa, myd88, il10, casp1, nos2a, tgfb1a, nfkb, tlr1, tlr2, tlr3*, and *tlr9* (also in protein level, expect for Tlr2).	–Upregulated *cnr1/2* and *abhd4* and downregulated *faah* and *mgll* in the gut compared to controls.	(81)
Probiotic	*Bacillus amyloliquefaciens*	Adult fish (30 days feeding)	–	–Upregulated expression of *il-1β, il6, il21, tnfa, lyspzyme, tlr1, tlr3, and tlr4*.	–Increased survival after *A. hydrophila* and *S. agalactiae* challenges.	(82)
Probiotic	*E. coli 40, E. coli Nissle*, and *E. coli MG 1655 ΔptsG*.	Adult zebrafish		–*E. coli 40* and *E. coli Nissle* decreased mucin found in water after *V. cholerae O395* or *V. cholerae El Tor* strain *N16961* challenge.	–	(83)
Probiotic & prebiotic	*Lactobacillus casei BL23* and exopolysaccharide-protein complex (EPSP)	3–12 dpf	–Microbiota did not change due to *L. casei BL23*.	–*L. casei* upregulated *tnfa, il-1β, il-10*, and *Saa* after 24 h infection with *A. veronii* but downregulated after 48 h. ESPS increased *tlr1, tlr2, il10, tnfa* expression, and decreased *il-1β* exp.	–*L. casei BL23* and EPSP increased survival after *Aeromonas veronii* infection.	(84)
Probiotic & prebiotic	*Ecklonia cava* (EC) Celluclast enzymatic EC (ECC) 100% ethanol extract EC (ECE).	Adult zebrafish (21 days feeding)	–E. cava induced *L. brevis, L. pentosus* and *L. plantarum* growth.	–EC combined with *L. plantarum* increased *iNOS* and *COX2* in the gut after *E. tarda* challenge.	–EC, ECC, and ECE diminished colony counts of *E. tarda, S. iniae*, and *V. harveyi*. EC reduced mortality *after E. tarda* challenge	(85)

Fucoidans extracted from several brown algae; *Eklonia cava* (64), *Chnoospora minima* (66), and *Turbinaria ornata* (65) were administrated to zebrafish larvae in the water. In all three studies, larvae exposed to fucoidans displayed reduced levels of reactive oxygen species (ROS), inducible nitric oxygen synthase (iNOS) and improved cell viability in whole larvae after LPS challenge (64–66). However, in these studies the candidate prebiotics were diluted in the water when the embryos were 8 h post-fertilization. Since the mouth of the zebrafish embryo does not open until 3 dpf and the complete digestive tract is not fully developed until 6 dpf (12) such studies do not prove a prebiotic effect on gut immunity. Preferably, zebrafish larvae with a fully developed digestive tract (6 dpf or older) are employed to study such interactions. Furthermore, prebiotics should be tested at physiologically relevant concentrations. Testing a prebiotic in zebrafish larvae may uncover a prebiotic function but often the overall goal would be to formulate novel diets containing the optimal concentration of prebiotic. For this aim, juvenile or adult zebrafish would be more suitable. We investigated the effect of fucoidan derived from the brown alga *Cladosiphon okamuranus* on microbiota composition in whole larvae (water exposure) and in adult zebrafish gut (feeding with flakes). In the gut of adult zebrafish, gene expression of *il-1*β was reduced and the dominant *Escherichia coli* (Proteobacteria) decreased in favor of Rhizobiaceae and Burkholderiaceae after feeding with fucoidan, while in larvae *il-1*β*, il-10, tnfb*, and *mmp9* increased but no microbial changes were observed (Ikeda-Ohtsubo, this issue).

Differently from fucoidans, β-glucans can act as immunostimulators in zebrafish. Beta-glucans from oats, upregulated gene expression of *tnfa, il-1*β*, il-10, il-12, defb1, lyz*, and *c-rel* in a dose-dependent manner in 5 dpf whole zebrafish larvae (67). In a similar study, β-glucan exposure from 4 hpf until 6 dpf upregulated *tnfa, mpo, tlf*, and *lyz* gene expression (68). In both studies, β-glucan administration in the water hampers its uptake quantification by the fish and again the exposure of very young larvae probably does not lead to gut-related effects. Oligosaccharides such as galactooligosaccharides (GOS) and fructooligosaccharides (FOS) are frequently used as prebiotics in agriculture and human infant nutrition to boost health via increased production of suggested beneficial bacterial fermentation products (63). Adult zebrafish fed with GOS for 8 weeks at 0.5, 1, and 2% inclusion levels displayed upregulation of *tnfa* and *lyz* expression and an increase in total immunoglobulins in the whole zebrafish (69). However, no gut specific read-outs were assessed.

It is clear that prebiotics can act on the immune system in a specific manner depending on their source of origin. Fucoidans can decrease inflammation markers whereas β-glucans and GOS increase gene expression of pro-inflammatory cytokines. Despite the promising outcomes, the vast majority of studies exposed undeveloped larvae to prebiotics which are unable to ingest the additive via free feeding. Prebiotics research should carefully evaluate gut health because is the organ where feed can potentially modulate the microbiota and the host immune system. If such candidate prebiotics are included within dry pellets and administrated to fish slightly before satiation (ensuring fish eat all the pellets), it is feasible to estimate the prebiotic gut levels and assess effects on gut microbiota and immunity with more clarity.

Several methods not yet extensively employed in the previously mentioned prebiotic studies may also be suitable for prebiotics gut health research in zebrafish. Firstly, histology and immunohistochemistry staining is needed to understand the immuno-modulatory effects in the gut tissue (i.e., disruption of the normal gut architecture). Transgenic zebrafish could potentially help to clarify which subpopulations of immune cells infiltrate the gut using fluorescently-activated cell sorting (FACS) and imaging. Furthermore, cell sorting of these sub-populations together with transcriptomics would depict the real effect of the prebiotic. Omics technologies (genomics, transcriptomics, proteomics, etc.) play an increasing important role in understanding the immune effects of aqua-feeds [reviewed in (86)] and omics-based read-outs should become more popular as their costs decrease.

Comparing the limited number of studies performed on zebrafish with a much larger number of studies performed in aquaculture species confirms that supplementation of β-glucans to feed of Atlantic salmon, trout or sea bass increases immune activity [reviewed in (87)] and trained immunity (88). However, only a limited number of studies have been performed on GOS supplementation. Dietary supplementation to Atlantic salmon of GOS at 1 g/kg feed for 4 months did not show effects on reactive oxygen species (ROS) production or lysozyme activity. Research on the use of seaweed is increasing, for example testing 10% inclusion levels of *Laminaria digitata* in feed of Atlantic salmon (89). The dietary seaweed improved chemokine-mediated signaling but the study only assessed transcriptional responses after LPS challenge so further research into the health effects of elevated or reduced gene expression is warranted. This last example nicely supports the use of zebrafish model, not to replace testing in aquaculture target species, but to prescreen feed components and further dissect the mechanism of action by live imaging and assessment of health parameters for prolonged periods, something difficult to achieve in large and costly aquaculture species.

## Probiotics

Already in 1907, Elie Metchnikoff related the use of probiotics to elongation of life expectancy. For the purpose of this review we define probiotics as a live or inactivated microorganism, such as bacterium or yeast, that when administrated via feed or water, confers a benefit to the host, such as improved disease resistance or enhanced immune responses [adapted from (90, 91)]. Probiotics can influence the health of the host in several ways: secreting secondary metabolites that inhibit growth of microbial pathogens and/or directly stimulating immune responses to downregulate gut inflammation (92). Here we focused on the probiotic studies in zebrafish concerning (gut) immune and microbiota modulation (summarized in Table 1).

To assess potential health benefits of live probiotics it is important to understand their optimal environment inside the host (oxygen levels, pH, etc.) and their colonization route. Probiotic-host interaction was addressed by a model of oro-intestinal pathogen colonization in GF zebrafish (76). Firstly, 6 dpf zebrafish were exposed by immersion to 25 potential enteric fish pathogens after which mortality was recorded during 3 days. *Edwardsiella ictaluri* caused the highest larvae mortality and was further selected to challenge the fish. Then, larvae were pre-colonized with single strains of 37 possible probiotics prior to *E. ictaluri* challenge. From this extensive screening, *Vibrio parahaemolyticus, E. coli* ED1a-sm and *E. coli* MG1655 F' provided a significant increase in survival upon *E. ictaluri* infection. *V. parahaemolyticus* protected the host by inhibiting *E. ictaluri* growth whereas *E. coli* protected via specific adhesion factors, such as F pili involved in biofilm and conjugation formations offering niches to other probiotic bacteria in the host (76). It is of note that zebrafish gills, although they are active in gas exchange 2 weeks after fertilization (93), provide a potential portal of entry for pathogens. Regretfully, gills were not included in the aforementioned study. Interestingly, in the same study, *Vibrio parahaemolyticus* was assessed as a possible probiotic whereas *Vibrio ichthyoenteri* was considered as a possible pathogen. The majority of the microbiota studies associate immune responses to taxonomic levels such as genera or families (i.e., *Vibrio* spp.) rather than species or strains. As a consequence, there is a generalization of an entire genus to a functions that could be species or even strain-specific. Such widely used generalizations may come from the difficulty to generate amplicons that are long enough to discriminate between closely related organisms. Besides, transcriptomics and shot gun approaches are preferred over 16S rRNA gene analysis to depict the active microbiota because they more informative regarding the fish health status (21). Adult zebrafish were also used to test probiotics as a model for human probiotic consumption. Adult zebrafish were exposed to two *E. coli* strains (Nissle and MG 1655 ΔptsG*)* and challenged with species of *Vibrio choleae* (strain El Tor)*. E. coli* spp. decreased the mucin content found in the tank water, indicator of diarrhea (83) although these mucins could perhaps also result from skin shedding. It might be interesting to assess whether these *E. coli* spp. increase secretory cell development and therefore mucus secretion via reduction of Myd88-Notch signaling as previously reviewed (39). In addition, while in humans administration of bacteria via a solutions orally ingested is an efficient way of ensuring ingestion, addition of probiotics to the water may not guarantee uptake by fish and may affect overall fish mucosa (skin, gills, gut) and not only uptake in the gut. Besides, the environment of the fish gut is more aerobic than the human gut environment (21) and lactic acid bacteria may be outcompeted by other bacteria in these aerobic conditions. This rationale may explain why human probiotics (*Lactobacillus* spp.) tested in zebrafish by immersion did not confer protection against *E. ictaluri* infection (76). Several studies reported Lactic Acid Bacteria (LAB) as good probiotic candidates due to their ability to withstand and adhere to the gut, their lactic acid production which inhibits the growth of pathogenic bacteria and their strengthening of the mucosal barrier (94). Zebrafish immersed with *Lactobacillus casei* BL23 from 3-25 dpf displayed an increased survival compared to controls after an immersion challenge with *Aeromonas hydrophila*. Gut gene expression of *il-1*β, *C3a*, and *il-10* was upregulated after 8 and 24 h after *A. hydrophila* challenge compared to controls (71). Interestingly, potential probiotics from the genera *Lactobacillus* modulated gene regulation in a strain-specific fashion. As a matter of fact, GF larvae immersed with *Lactobacillus fermentum* NA4 displayed an increased *il-10* expression and a decreased *il-1*β and *tnfa* expression after chemically-induced inflammation compared to controls. However, in the same study, larvae immersed with several strains of *Lactobacillus plantarum* (WCFS1 and NA7] or other *Lactobacillus fermentum* strains (ATCC9338 and NA6) did not show these differences in gene expression (75). Dissimilarities in gene expression among the aforementioned studies (71, 75) could be due to fish age (3–25 vs. 7 dpf), tissue analyzed (gut vs. whole larvae) challenge applied (live pathogen vs. chemical) and the specific *Lactobacillus* strain used as a probiotic candidate. *Bacillus amyloquefaciens* supplemented twice a day for 30 days in a commercial diet upregulated *il-1*β*, il-6, il-21 tnfa, lysozyme, tlr1, tlr3*. and *tlr4* expression in adult zebrafish whole body and increased survival during *A. hydrophila* and *S. agalactiae* challenge (82). Upregulation of gene expression appeared related to enhanced innate immunity although no other immune parameters were taken into account. In another study in adult zebrafish, a commercial diet was supplemented with multiple lyophilized probiotic strains for 30 days. The probiotic mix upregulated *il-1*β*, tnfa, myd88, il-10, casp1, nos2a, tgfb1a, nfkb, tlr1, tlr2, tlr3*, and *tlr9* expression in the gut. Furthermore, the probiotic mix increased the protein levels encoded by all the upregulated genes (except for Tlr2 protein) (81). On the one hand, certain bacteria of the probiotic mix may have inhibited Tlr2, which in turn could have partly suppressed *myd88* (38). On the other hand, other bacteria of the probiotic mix may have enhanced expression of other TLRs that upregulated *myd88* and the overall Myd88-balance orchestrated innate immune responses. As previously reviewed, microbial species can influence host immunity irrespective of their abundance (53) and when using mix of probiotics the effects of each individual species are harder to disentangle. Other studies using LAB as probiotics did not only examined gene expression but also microbiota (73, 77, 79) and histological changes (77, 78) in the zebrafish gut (Table 1). Some studies investigated the potential of yeast as a probiotic for zebrafish. GF and CONV zebrafish larvae were immersed from 2–3 dpf in solutions of two yeasts after which gut microbiota were sampled at 14 dpf (70). Although microbial changes were observed, immune-related outcomes where not measured so the probiotic effect of the yeasts in this study remains undefined. In another study, 4 dpf zebrafish were exposed to 15 fluorescently labeled yeast strains for 2 h prior to *Vibro anguillarum* challenge (74). Most of the yeast strains conferred increased survival after challenge. In a later experiment, the same group further studied two of the yeast strains in GF and CONV larvae using a similar set-up. Exposure to either yeast strain significantly increased survival in GF and CONV larvae after *V. anguillarum* challenge (72). CONV zebrafish challenged with *V. anguillarum* displayed an upregulation of *il-1*β*, c3, tnfa, mpx*, and *il-10* expression. Pre-treatment with either yeast strain prevented such gene upregulation in CONV and GF larvae, indicating that these yeast strains might prevent or reduce the effects of *V. anguillarum* (72).

Zebrafish have also been employed for synbiotic studies which typically combine the use of prebiotics and probiotics. *Lactobacillus casei* BL23 and an exopolysaccharide complex (ESPS) were studied in combination in GF and CONV larvae from 3 to 12 dpf. *L. casei* exposure upregulated *tnfa, il-1*β*, il-10*, and *saa* expression after 24 h in a challenge with *Aeromonas veronii* and downregulated expression of these genes after a 48 h challenge. It is of note that the ESPS alone upregulated *tlr1, tlr2, il-10*, and *tnfa* and downregulated *il-1*β after 24 h challenge. Synbiotically, *L. casei* BL23 and EPSP improved survival dose-dependently after *A. veronii* challenge (84). The combined supplementation of *E. cava* enzymatic digest, with enhanced biological activity, as prebiotic together with *L. plantarum* as a probiotic in adult zebrafish for 21 days reduced the level of *iNOS* and *cyclooxygenase 2* (*cox2*) in the gut. Moreover, when prebiotics and probiotics were administrated together, they increased survival compared to *L. plantarum*-treated fish alone after a challenge with *E. tarda* (85). Interestingly these studies suggest that certain extracts and/or biologically active compounds rather than the whole prebiotic may cause immune-modulation.

**Figure 2:**
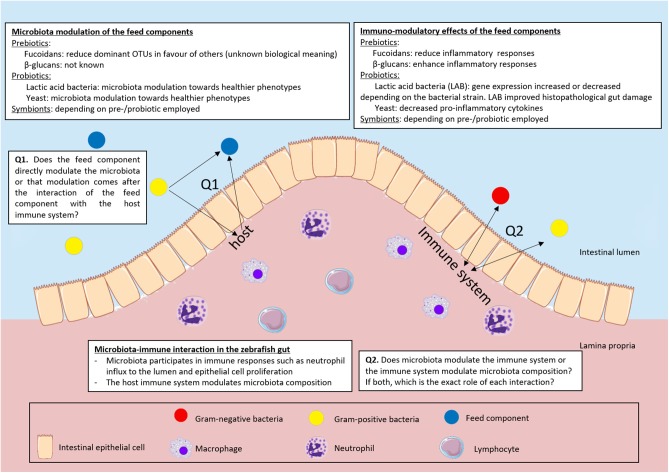
Overview of the interaction of pre- and probiotics, immune system and microbiota in the zebrafish intestine. We summarized the interactions of microbiota and feed components, immune system and feed components and microbiota and immune system. We highlighted the questions that still remain unsolved in the field.

A large number of studies (co)exposed potential prebiotics and/or probiotics to zebrafish to improve their immune condition via microbial modulation (Figure 2). Remarkably, in most of these studies, gene expression was assumed a conclusive immunological read-out. Apart from the fact that gene expression does not always translate to protein functionality, often pro- and anti-inflammatory cytokines are upregulated or downregulated depending on the dynamics and the timing of the response. The gene expression may reflect the balance in the host during an immune response: specific and strong enough to fight potentially pathogenic bacteria but at the same time able to tolerate commensal host microbiota (95). This balance is also dependent on different cell types that work in concert to prevent excessive damage to the host when acting against an invading pathogen or ongoing inflammation. We need to understand the role and presence of different immune cell types that are involved in the different responses in much more detail before we can try to modulate the response to the benefit of the host. To this end, the zebrafish remains the ideal candidate model organism. To date, more studies could have made use of the unique tools in zebrafish such as live imaging of different transgenic reporter zebrafish (cytokines as well as immune cell populations) to get a much broader understanding of the complex dynamic interactions of host-feed-microbe interactions.

## Concluding Remarks

In this review we focused on the zebrafish as an animal model to study the effect of feed on host-microbe-immune interactions (summarized in Figure 2). Zebrafish are now widely used as models to study fundamental and evolutionary processes that might uncover pathways relevant for both fish and mammals. The studies on microbial composition development summarized in this review reveal that although the gut microbial composition is dependent on salinity, trophic level and host phylogeny, mammalian, fish and insect gut microbiota still cluster together and separately from environmental samples. Thus, although mammals and fish live in distinct environments and clearly have different physiology, gene expression and regulation of gene expression in the gut is highly similar. IEC transcriptional profiles are more similar between species than responses of different cell types of the same species. Therefore, experimentation with zebrafish seems suitable to elucidate conserved molecular mechanisms.

Using zebrafish as a model for aquaculture species is of interest. Eighty percent of farmed fish are other cyprinids and therefore close relatives. We argue that using the zebrafish as a model for aquaculture species brings several advantages yet may never fully replace studies performed in the target species for validation. Nevertheless, using zebrafish as a pre-screen model to guide studies in aquaculture species might contribute to elucidate mechanisms underlying feed and host-microbe-immune interactions.

Recently, exiting new research using *in vivo* mice models has shown that the microbial community can influence the severity of viral infections (96, 97). Moreover, *in vitro* data using RAW264.7 cells showed antiviral activity of several *Lactobacillus* strains to murine norovirus (MNV) infection through IFN-β upregulation (98). Currently, it is unknown whether microbes can also alter fish-specific viral infectivity. This is an exciting new avenue of research that might lead to novel vaccination strategies, combining virus-targeting vaccines with prebiotic or probiotic treatment to change the microbiota as well as target the virus itself. A fundamental field in which zebrafish are most probably will contribute due to its unique advantages.

The studies published in the field using zebrafish will continue to increase and by combining existing technologies (omics, immunohistochemistry, FACS, *in vivo* imaging) or by emerging novel technology knowledge gaps will surely be filled. For future experiments it would greatly benefit our understanding if more holistic approaches would be taken. We need to combine read-out parameters such as gene expression, survival after challenges, gut architecture, immune cell recruitment, microbiota composition, metabolite production and behavioral data within each experiment to provide a broader picture of the consequences of certain treatments on the health of the fish. Only by carefully determining cause and effect by interrogating possible molecular pathways through gene editing we can provide a solid rationale for the design of novel immunomodulatory strategies.

## Author Contributions

AL drafted the manuscript and the figures. WI-O, DS, DP, CM, MF, GW, and SB edited and contributed to writing the manuscript. SB, GW, and DS obtained the funding.

### Conflict of Interest

DP and CM are employed by Skretting Aquaculture Research Center. The remaining authors declare that the research was conducted in the absence of any commercial or financial relationships that could be construed as a potential conflict of interest.
